# A machine learning approach to predicting dry eye-related signs, symptoms and diagnoses from meibography images

**DOI:** 10.1016/j.heliyon.2024.e36021

**Published:** 2024-08-13

**Authors:** Andrew D. Graham, Tejasvi Kothapalli, Jiayun Wang, Jennifer Ding, Vivien Tse, Penny A. Asbell, Stella X. Yu, Meng C. Lin

**Affiliations:** a Department of Electrical Engineering and Computer Sciences, University of California, Berkeley, United States; b Vision Science Group, University of California, Berkeley, United States; c Clinical Research Center, School of Optometry, University of California, Berkeley, United States; d Department of Bioengineering, University of Memphis, United States; e International Computer Science Institute, Berkeley, United States

**Keywords:** Machine learning, Artificial intelligence, Meibography, Meibomian gland morphology, Dry eye, Meibomian gland dysfunction, Ocular surface

## Abstract

**Purpose:**

To use artificial intelligence to identify relationships between morphological characteristics of the Meibomian glands (MGs), subject factors, clinical outcomes, and subjective symptoms of dry eye.

**Methods:**

A total of 562 infrared meibography images were collected from 363 subjects (170 contact lens wearers, 193 non-wearers). Subjects were 67.2 % female and were 54.8 % Caucasian. Subjects were 18 years of age or older. A deep learning model was trained to take meibography as input, segment the individual MG in the images, and learn their detailed morphological features. Morphological characteristics were then combined with clinical and symptom data in prediction models of MG function, tear film stability, ocular surface health, and subjective discomfort and dryness. The models were analyzed to identify the most heavily weighted features used by the algorithm for predictions.

**Results:**

MG morphological characteristics were heavily weighted predictors for eyelid notching and vascularization, MG expressate quality and quantity, tear film stability, corneal staining, and comfort and dryness ratings, with accuracies ranging from 65 % to 99 %. Number of visible MG, along with other clinical parameters, were able to predict MG dysfunction, aqueous deficiency and blepharitis with accuracies ranging from 74 % to 85 %.

**Conclusions:**

Machine learning-derived MG morphological characteristics were found to be important in predicting multiple signs, symptoms, and diagnoses related to MG dysfunction and dry eye. This deep learning method illustrates the rich clinical information that detailed morphological analysis of the MGs can provide, and shows promise in advancing our understanding of the role of MG morphology in ocular surface health.

## 1 Introduction

Dry eye (DE) is a highly prevalent condition affecting ocular surface health, vision, and quality of life for millions of people [1,2] and is the reason for the majority of eye care clinical visits [3]. The most common manifestation of DE is evaporative [2] and the primary causative factor is thought to be Meibomian gland dysfunction (MGD) [4,5]. In many cases of MGD, the glands are unable to secrete a sufficiently thick and uniform lipid layer, allowing the aqueous tears to evaporate and leading to rapid tear film thinning and destabilization, hyperosmolarity, tear film breakup, and ultimately to DE symptoms [4,6]. Alth ough changes in the morphology of the Meibomian glands (MG) are presumed to be the primary mechanism of MGD, there has been little investigation into the detailed morphological characteristics of the MG or their role in ocular surface pathology and the signs and symptoms of DE.

Infrared meibography provides visualization of the MG of an everted eyelid. Currently, a visual estimation of the % area of gland atrophy from meibography remains the most widely employed index for characterizing gland morphology. Attempts have been made to manually quantify the structures of the individual glands (e.g., length, width, tortuosity) [[7], [8], [9]]. These methods are laborious, have poor reproducibility, are subject to human bias, cannot be performed in a timely manner in a clinical care setting, and are not suitable for processing large numbers of meibography images for research purposes.

The use of artificial intelligence (AI) in medical imaging is rapidly expanding; however, the application of the technology to ocular surface health care and research remains sparse [[10], [11], [12], [13], [14], [15], [16]]. A few studies have demonstrated the power of AI to efficiently and quantitatively characterize MG features in detail, primarily by segmenting individual MG from meibography images and quantifying various global (eyelid level) and local (individual gland level) morphological features [17,18]. However, the impact of gland features on the downstream signs and symptoms of MGD and DE has yet to be extensively explored.

In the present work, a novel interpretable deep learning algorithm was developed to predict signs, symptoms, and diagnoses using meibographic imaging. A previously published supervised segmentation and attribute learning model [10] first quantifies a range of MG morphological characteristics from meibography images. These metrics are then combined with corresponding clinical assessments of the ocular surface, eyelids and tear film, and symptom questionnaire responses in prediction models of MGD- and DE-related outcomes.

## 2 Methods

### 2.1 Subject recruitment

Subjects were recruited from the University of California, Berkeley campus and surrounding community. Eligible subjects included contact lens wearers and non-wearers at least 18 years of age. Eligible contact lens wearers discontinued lens wear 24 h prior to study visit. Exclusion criteria included currently active ocular infection or inflammation, ocular surgery in the previous 6 mo, and females pregnant or nursing. This study adhered to the tenets of the Declaration of Helsinki and was approved by the UC Berkeley Committee for the Protection of Human Subjects (Approval #2010-02-792). Informed consent was obtained from all participants. This study conformed to CONSORT-AI Extension guidelines for clinical studies with an AI component [19].

### 2.2 Meibomian Gland morphological characteristics

Meibography images of both eyes were captured with the OCULUS Keratograph 5M (OCULUS, Arlington, WA). A total of 458 images were used in the analysis. Each meibography image was input to a supervised image segmentation and attribute learning model to differentiate individual glands in the image and to quantify local (gland-level) and global (eyelid-level) morphological characteristics. The learned morphological characteristics consisted of gland length, width, tortuosity, local contrast, number of visible glands, gland density, % area of gland atrophy, and percentage of ghost glands. These morphological features were then merged with data from clinical assessments and questionnaire responses from instruments designed and validated for ocular discomfort and DE (detailed below).

### 2.3 Questionnaires

Instruments to collect data on subject characteristics, contact lens wear histories, and ocular symptomatology were administered at the beginning of a single day visit. Validated DE questionnaires included the Ocular Surface Disease Index (OSDI) [20], the Standard Patient Evaluation of Eye Dryness (SPEED II) [21], the Berkeley Dry Eye Flow Chart (DEFC) [22], the 8-item Contact Lens Dry Eye Questionnaire (CLDEQ-8) for lens wearers [23], and the 5-item Dry Eye Questionnaire for non-wearers (DEQ-5) [24]. In addition, subjects completed Visual Analog Scale (VAS) ratings (0–100) [25] of ocular discomfort and dryness frequency and severity throughout the day and at end-of-day.

Presenting a large set of questionnaires in a non-random order would not be advisable as whichever instrument is presented last would always be completed by subjects in their most fatigued, bored, distracted, or impatient state. Rather than randomizing, the symptom questionnaires were presented in an order determined by constructing a Williams Pair [26,27]. This technique is a useful alternative to standard randomization when the number of possible questionnaire (or other “treatment”) orderings far exceeds the number of subjects and all subjects are being administered all questionnaires, as it balances assignments over time and prevents any more than two consecutive subjects from having the same ordering.

### 2.4 Ocular signs

Clinical assessments of the eyelids, cornea, conjunctiva, and tear film were performed by a team of trained and certified research optometrists after completion of the questionnaires, in a sequence designed to minimize carryover effects from one clinical test to the next. Tear lipid layer thickness and variability were measured with the LipiView interferometer (TearScience, Morrisville, NC, USA), followed by tear volume measurement at the lower meniscus and grading of bulbar and limbal hyperemia using the Oculus Keratograph 5M (OCULUS, Arlington, WA, USA). The Medmont E300 corneal topographer (Medmont International PTY LTD, Nunawading, Australia) and a stopwatch were used to measure non-invasive tear breakup time (NITBUT), followed by clinical grading of conditions of the eyelids and lashes. Sodium fluorescein dye (1 μl of 1 % solution) was instilled next for slit lamp measurement of fluorescein tear breakup time (FTBUT), followed by grading of corneal staining. The Meibomian Gland Evaluator [28] (TearScience, Morrisville, NC, USA) was then used to express meibum for quantity and quality assessment, followed by instillation of lissamine green dye (10 μl from 5 drops of saline solution and 3 dye strips fully saturated) [29] and grading of conjunctival staining. Corneal and conjunctival staining were graded according to CCLRU grading scales (Brien Holden Vision Institute, Sydney, Australia). Meibum quality was quantified by assigning each gland a score ranging from 0 (no secretion) to 3 (clear liquid secretion), multiplying the number of glands by their respective scores and summing. Meibum quantity was similarly graded, with quantity scores from 0 (complete blockage of the gland orifice) to 3 (copious meibum expressed). Grades were assigned over the entire exposed tarsal plate, and separately for the central 50 % of the tarsal plate. The eyelids were then everted for meibography imaging using the Oculus, and finally aqueous volume was measured with the Schirmer I test without anesthetics.

### 2.5 Diagnoses

Prior to the study, a focus group was formed to standardize binary (yes/no) clinical diagnoses of MGD, aqueous deficiency, blepharitis and lagophthalmos. MGD was defined by ductal stenosis and cloudy or inspissated quality of expressed meibum. Aqueous deficiency was diagnosed by a Schirmer test strip wetted length of <5 mm at 5 min without anesthesia. A diagnosis of blepharitis was based on the presence of eyelid margin inflammation, debris, and collarettes. Lagophthalmos was diagnosed based on transilluminating the eyelid and observing light escaping from the aperture of an incompletely closed lid. The group consisted of the lead clinical investigator (MCL) and three experienced optometrists. Previously collected subject records were reviewed and independent diagnoses made in order to calibrate all observers to the same criteria. Diagnostic criteria were standardized after repeated records reviews and subsequent discussions with the lead clinical investigator. Once concordance among the clinicians was achieved, investigators made clinical diagnoses of the aforementioned diseases/conditions upon completion of the study visit for each subject.

### 2.6 Prediction model input and output, data pre-processing

Three types of predictions were made for this study: (1) clinical signs, (2) subjective symptoms, and (3) diagnoses. Subject characteristics such as demographics and contact lens histories were available as potential predictive features for all models. Prediction models were initially run with all clinical, subjective, and subject characteristic variables available as potential predictors, and again using only the 8 machine learning-quantified MG morphological characteristics as potential predictive features. Image processing techniques and dataset selection criteria are detailed elsewhere [[10], [11], [12]]. Data were pre-processed to remove variables that contained little or no information. Sparse variables were removed, such as those with a <1 % positivity rate (e.g., only 0.7 % of subjects presented with gout) and variables with <5 % response rate.

### 2.7 Prediction model training and validation

**Fig. 1:**
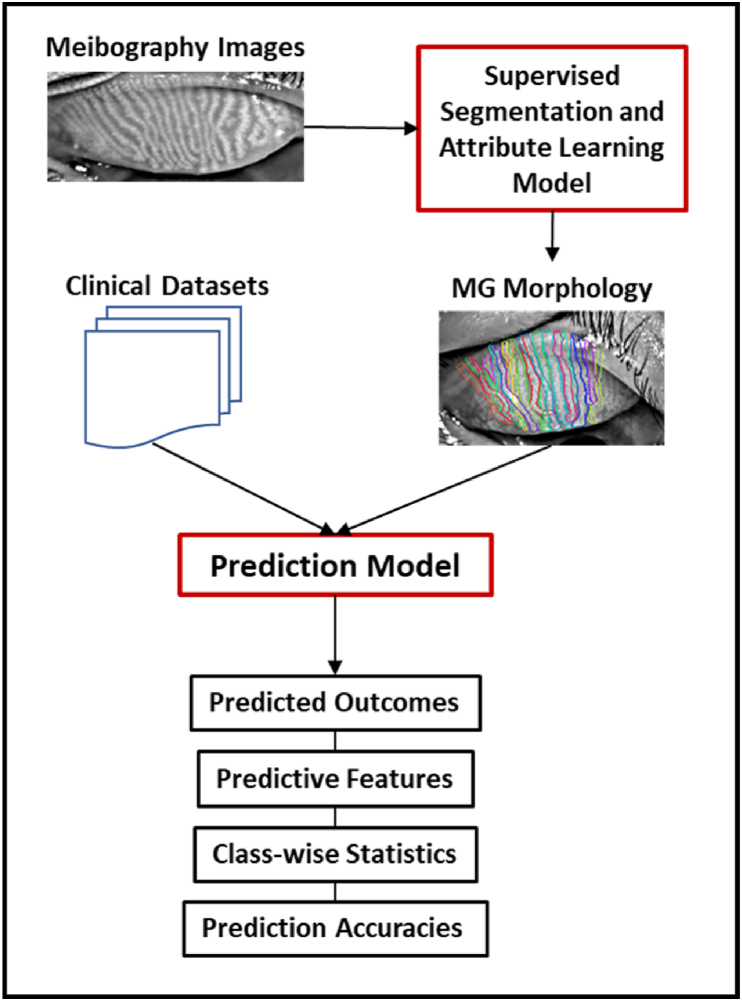
The overall pipeline including metrics derived from meibography images combined with clinical datasets to make ocular surface disease-related outcome predictions.

The overall pipeline for predicting DE-related outcomes is diagrammed in Fig. 1. For a given outcome prediction, the MG morphological characteristics learned from the segmentation and attribute learning model and corresponding clinical and subjective data were randomly allocated into 5 training and validation subsets. The model was trained on 4 of the 5 subsets with the 5th subset being used for validation. The model was first trained on all available features, the prediction accuracy recorded, then the lowest weighted feature was pruned and the model run again on the remaining features; the process was repeated until only a single feature remained and the model with the highest prediction accuracy was identified. In order to mitigate the chances of a spurious or non-generalizable prediction due to distributional mismatch between the training data and a single randomly selected validation set, the process was repeated with each of the 5 subsets being used as the validation set and the results from all 5 best-accuracy models combined. To generate the final output, the coefficients from the 5 best-accuracy models were aggregated and ranked, and the five features with the largest coefficient values (i.e., the most heavily weighted features used for the predictions), the mean accuracy, and the median number of features were recorded. The prediction model employed logistic regression for classification into outcome classes, and used an L2 regularization penalty term to avoid over-fitting. A limited-memory Broyden-Fletcher-Goldfarb-Shanno (L-BFGS) algorithm was employed as it is well-suited to solving problems with many variables. Training iterations were set to 500. All training, validating and testing was performed on a single NVIDIA GeForce GTX 2080 GPU. The processing time per meibography image was approximately 0.5 s.

One further step was required for the final output: the calculation of the class-wise statistics of the most heavily weighted features for each predicted class of the outcome. Logistic regression was employed by the algorithm to predict outcomes into one of two or more ordinal classes. Categorical outcomes (e.g., MGD diagnosis [No/Yes]; eyelid notching [Absent/Present]) have natural predicted classes; for continuous and ordinal outcomes (e.g., tear breakup time [sec]; corneal staining [0–3 grade]), predicted classes were based as much as possible on published thresholds in the literature, and on clinical experience and standard practice where no published guidelines exist. Table 1 shows the predicted classes for each outcome, along with the sources upon which these classes were based where available. Overall, the performance of each model is assessed by examining the 5-fold cross-validation accuracy, the class-wise statistics, and the clinical interpretability of the most heavily weighted predictive features.

**Table 1:** Predicted classes and thresholds for ordinal and continuous outcome variables. Classes were based to the extent possible on published thresholds, and where such established numbers were not available, on extrapolations from relevant literature and clinical experience.

Outcome	Data Type	Predicted Classes	Source
**Clinical Signs**			

Blepharitis	Clinical grade, 0-3	[<2, ≥2]	–
Lid Margin Erythema	Clinical grade, 0-3	[<2, ≥2]	–
Line of Marx Displacement	Clinical grade, 0-3	[<2, ≥2]	–
Lid Wiper Epitheliopathy, Length	Clinical grade, 0-3	[<2, ≥2]	–
Lid Wiper Epitheliopathy, Width	Clinical grade, 0-3	[<2, ≥2]	–
Meibum Quality & Quantity, Central	Assessment, 0-45	[≤18, >18]	Cochenor, 2018
Meibum Quality & Quantity, Entire	Assessment, 0-90	[≤36, >36]	ibid., by extension
# of Visible MG, Upper Eyelid	Count	[<10, ≥10 < 20, ≥20]	Arita, 2009; Butovich, 2017
# of Visible MG, Lower Eyelid	Count	[<8, ≥8 < 17, ≥17]	ibid.
Tear Lipid Layer Thickness (nm)	Continuous	[≤60, >60]	Isreb, 2003
Tear Lipid Layer Variability (CV)	Continuous	[≤0.052, >0.052]	Lin, 2016
Non-invasive Tear Breakup Time (s):			
All Subjects	Continuous	[<10.0, ≥10.0]	–
Asian	Continuous	[<9.0, ≥9.0]	Yeh, 2015
Non-Asian	Continuous	[<10.0, ≥10.0]	Yeh, 2015
Fluorescein Tear Breakup Time (s):			
All Subjects	Continuous	[<10.0, ≥10.0]	–
Asian	Continuous	[<6.7, ≥6.7]	Yeh, 2015
Non-Asian	Continuous	[<9.2, ≥9.2]	Yeh, 2015
Corneal Staining	Clinical grade, 0-3	[<2, ≥2]	Leonardi, 2018
Conjunctival Staining	Clinical grade, 0-3	[<2, ≥2]	ibid., by extension
Tear Meniscus Height (mm)	Continuous	[<0.25, ≥0.25]	Doughty, 2002
Schirmer Strip Length (mm)	Continuous	[<5.0, ≥5.0]	Danio, 1997

**Subjective Symptoms**			

OSDI	Continuous	[<12, >12 ≤ 23, >23]	Asiedu, 2016
SPEED II	Continuous	[≤4, >4]	ibid.
VAS Ratings			
Average Comfort	Continuous	[<75, ≥75 < 83, ≥83]	Li, 2016
Average Frequency of Discomfort	Continuous	[<10, ≥10 < 17, ≥17]	ibid.
Average Dryness	Continuous	[<20, ≥20 < 43, ≥43]	Graham, 2018
Average Frequency of Dryness	Continuous	[<19, ≥19 < 48, ≥48]	ibid.
EOD Comfort	Continuous	[<59, ≥59 < 76, ≥76]	Li, 2016
EOD Frequency of Discomfort	Continuous	[<17, ≥17 < 32, ≥32]	ibid.
EOD Dryness	Continuous	[<31, ≥31 < 61, ≥61]	Graham, 2018
EOD Frequency of Dryness	Continuous	[<32, ≥32 < 65, ≥65]	ibid.
DEQ-5	Continuous	[<6, ≥6 < 12, ≥12]	Chalmers, 2010
CLDEQ-8	Continuous	[<12, ≥12]	ibid.

* CV = Coefficient of Variation; EOD = End of Day.

## 3 Results

**Fig. 2:**
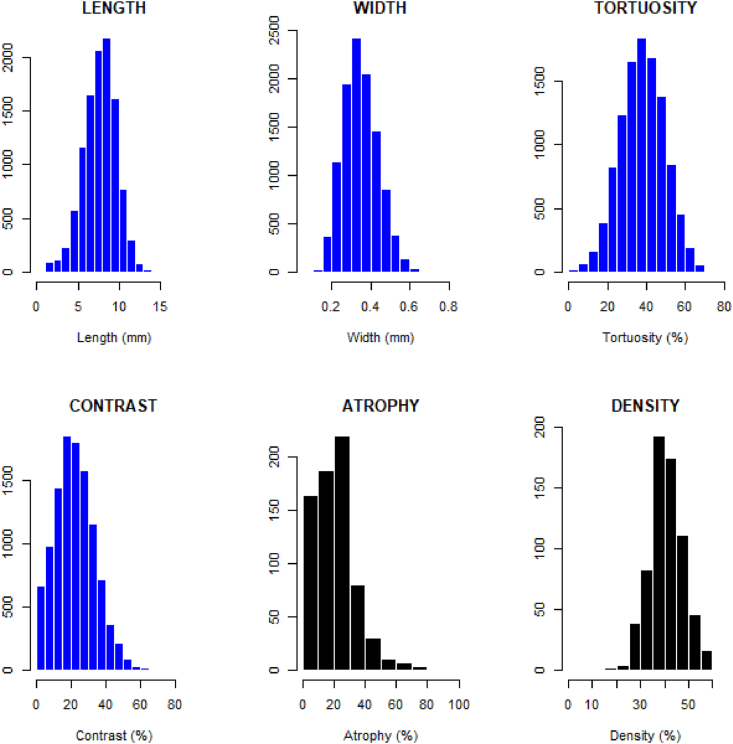
The distributions of quantified Meibomian gland morphological characteristics. Local gland-level features are shown in blue, global eyelid-level features in black. (For interpretation of the references to colour in this figure legend, the reader is referred to the Web version of this article.)

Subjects who completed the study were 67.2 % female and were 45.2 % Asian, 54.8 % Caucasian. Ages ranged from 18 to 71 yrs, with a mean (SD) of 26.6 (12.1) yrs. Contact lens wearers made up 46.8 % of the study sample. The first three sections below present models for the predictions of clinical signs, symptoms, and diagnoses, respectively. All available features, including subject demographics, MG morphological characteristics, clinical assessments and subjective symptom scores were available to the models as potential predictive features. In the fourth section, signs, symptoms, and diagnoses are predicted using MG morphological characteristics alone, without corresponding clinical and subjective data. The distributions of quantified MG morphological characteristics are presented in Fig. 2.

### 3.11 predicted outcomes: clinical signs

**Fig. 3:**
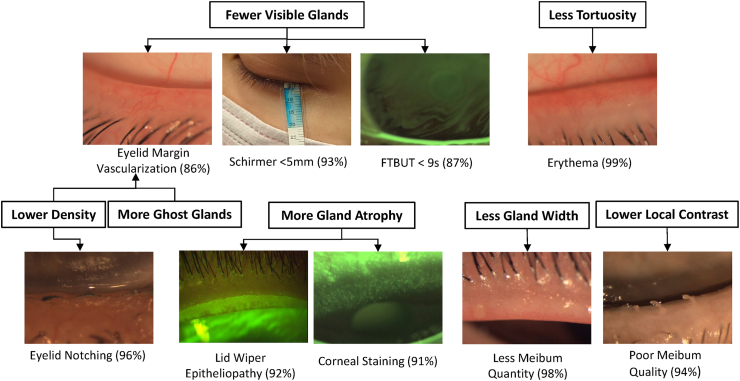
Machine learning models were able to predict various clinical signs using meibography and clinical data with over 90 % accuracy.

Machine learning-derived MG morphological characteristics were among the most heavily weighted predictors for 9 different clinical signs (Table 2). Overall, signs were predicted with accuracies ranging from 72.6 % to 99.1 %. Most clinical signs were predicted with accuracies above 90 % (Fig. 3).

**Table 2:** Prediction models for clinical signs with machine learning-derived MG morphology among the most highly weighted features. Shown are the statistics of each feature stratified on the predicted outcome classes, median number of features used and mean prediction accuracy.

Predicted Outcome [Predicted Classes]	Predictive Features	Class-wise Statistics	Total # Features	Prediction Accuracy (%)
**Predicted Outcomes: Clinical Signs**				

Eyelid Notching [Absent, Present]	MG Density (%)	[0.36, 0.31]	42	95.9
	Age (yrs)	[27.1, 46.7]		
	VAS EOD Dryness Freq (0–100)	[28.1, 38.4]		
Erythema: LL [<2, ≥2]	MG Atrophy Score: UL (0–3)	[0.68, 1.24]	60	99.1
	MG Tortuosity (%)	[31,36]		
Eyelid Vascularization [Absent, Present]	Ghost MG (%)	[6,15]	82	85.9
	MG Density (%)	[34,37]		
	Visible MG: UL (#)	[18.4, 16.0]		
	Erythema: UL (0–3)	[0.14, 0.95]		
Meibum Quantity: UL, Central [<18, ≥18]	MG Width (mm)	[0.32, 0.35]	38	98.0
	MG Atrophy Score: UL (0–3)	[0.71, 0.19]		
	Meibum Quality UL, Central (0-45)	[6.7, 22.0]		
	LWE Length (0–3)	[0.77, 0.06]		
Meibum Quality: LL, Entire [<36, ≥36]	MG Local Contrast (%)	[19,23]	16	94.0
	Meibum Quantity UL, Entire (0-90)	[12.9, 29.0]		
	Meibum Quantity LL, Entire (0-90)	[11.1, 34.4]		
Corneal Staining Extent [<2, ≥2]	MG Atrophy Area (%)	[17,23]	71	91.2
	VAS EOD Discomf Freq (0–100)	[27.9, 37.4]		
Tear Meniscus Height (mm) [<0.25, ≥0.25]	Ghost MG (%)	[7,11]	31	72.6
	NITBUT (sec)	[9.2, 15.1]		
	FTBUT (sec)	[6.5, 10.3]		
	PAS (mm)	[9.5, 10.2]		
FTBUT: Non-Asian (sec) [<9.2, ≥9.2]	Visible MG: UL (#)	[18.8, 21.3]	18	87.4
	NITBUT (sec)	[8.04, 18.94]		
	MG Atrophy Score: UL (0–3)	[0.71, 0.43]		
Schirmer Strip Length (mm) [<5.0, ≥5.0]	Visible MG: LL (#)	[13.3, 15.4]	36	92.5
	Conjunctival Staining (0–3)	[2.39, 1.53]		
	SPEED Score (0-28)	[7.94, 6.17]		

MG = Meibomian Gland; EOD = End-of-Day; UL = Upper Lid; LL = Lower Lid; LWE = Lid Wiper Epitheliopathy; CLW = Contact Lens Wear.

MG morphological characteristics were among the most heavily weighted predictors of gland function. Greater meibum quantity was predicted by greater gland width, lower atrophy score, better meibum quality and less lid wiper epitheliopathy (98.0 % accuracy). Better meibum quality was predicted by higher local contrast and greater meibum quantity (94.0 % accuracy).

Lower gland density was a heavily weighted predictor for eyelid notching (95.9 % accuracy) and eyelid margin vascularization (85.9 % accuracy). Greater age was also a heavily weighted predictor for eyelid notching, with a 19.6 yr mean age difference between those with and without notching. A higher percentage of ghost glands was an important predictor of eyelid margin vascularization, and of lower tear meniscus height (72.6 % accuracy) which model also included more tear film instability (i.e., shorter NITBUT and FTBUT).

Extent of corneal staining was predicted by greater % area of gland atrophy, along with a higher end-of-day frequency of discomfort VAS rating (91.2 % accuracy). Clinical outcomes predicted due in part to having fewer visible MG included eyelid margin vascularization, Schirmer strip wetted lengths <5 mm (92.5 % accuracy), and FTBUT <9.2 s among non-Asians (87.4 % accuracy). The prediction model for shorter Schirmer strip wetted lengths also included more conjunctival staining and a higher SPEED II score as heavily weighted predictors. Predictive features for shorter FTBUT among non-Asians also included shorter NITBUT and a higher MG atrophy score.

### 3.22 predicted outcomes: subjective symptoms

Machine learning-derived MG morphological features were among the most heavily weighted predictors for a number of subjective symptoms (Table 3), albeit with generally lower prediction accuracies for symptoms (60.7 %–86.5 %) than those achieved for clinical signs (72.6 %–99.1 %). The highest accuracies were achieved with the DEFC. DEFC assessment of the presence of any DE symptoms (mild to severe, inclusive) was predicted by less MG tortuosity (although only ∼1 % less), some small difference in gland density (<1 %) detectable by the algorithm but not clinically visible, and by a greater extent of corneal staining (76.8 % accuracy). DEFC debilitating symptoms predictions among non-contact lens wearers heavily weighted gland tortuosity, with approximately 5 % more tortuosity among asymptomatic subjects (86.5 % accuracy).

**Table 3:** Prediction models for subjective symptoms with machine learning-derived MG morphology among the most highly weighted features. Shown are the statistics of each feature stratified on the predicted outcome classes, median number of features used and mean prediction accuracy.

Predicted Outcome [Predicted Classes]	Predictive Features	Class-wise Statistics	Total # Features	Prediction Accuracy (%)
**Predicted Outcomes: Subjective Symptoms**				

OSDI [<12, ≥12 < 23, ≥23]	Visible MG: UL (#)	[19.7, 18.9, 17.5]	54	68.1
	FTBUT (sec)	[8.4, 9.8, 5.8]		
	Comfortable CLW (hrs/day)	[9.0, 8.2, 7.8]		
VAS Rating: Comfort [<75, ≥75 < 83, ≥83]	Ghost MG (%)	[5,12,14]	46	65.4
	Conjunctival Staining (0–3)	[2.1, 1.2, 1.3]		
	Palpebral Aperture Size (mm)	[9.7, 9.6, 10.0]		
	Comfortable CLW (hrs/day)	[7.5, 8.8, 9.3]		
VAS Rating: Dryness [<20, ≥20 < 43, ≥43]	Visible MG: UL (#)	[19.4, 19.8, 18.0]	46	66.1
	Age (yrs)	[25.9, 28.0, 32.8]		
	Comfortable CLW (hrs/day)	[9.2, 8.2, 7.7]		
DEFC Debilitating Symptoms: CLW [ASYM, CLIDE, DE]	Visible MG: LL (#)	[15.5, 15.7, 14.2]	46	63.9
	Visible MG: UL (#)	[17.8, 18.0, 18.1]		
	Comfortable CLW (hrs/day)	[11.8, 8.1, 7.6]		
DEFC Any Symptoms: Non-CLW [ASYM, SYM]	MG Density (%)	[0.36, 0.36]	24	76.8
	MG Tortuosity (%)	[0.36, 0.35]		
	Corneal Staining Extent (0–3)	[0.42, 0.72]		
DEFC Debilitating Symptoms: Non-CLW [ASYM, SYM]	MG Tortuosity (%)	[0.37, 0.32]	44	86.5

MG = Meibomian Gland; UL = Upper Lid; LL = Lower Lid; CLW = Contact Lens Wear; ASYM = Asymptomatic; SYM=Symptomatic; CLIDE=Contact Lens Induced Dry Eye.

### 3.33 predicted outcomes: diagnoses

Fewer visible MG was the only AI-derived morphological characteristic that contributed to predictions of clinician diagnoses (Table 4). The prediction model for MGD achieved an accuracy of 74.4 %. The five most heavily weighted features for a diagnosis of MGD were fewer visible glands, shorter NITBUT, a thinner lipid layer, fewer years of contact lens wear, and curiously a slightly lower OSDI score (although below the threshold of least clinically significant difference) [30].

**Table 4:** Prediction models for clinical diagnoses with machine learning-derived MG morphology among the most highly weighted features. Shown are the statistics of each feature stratified on the predicted outcome classes, median number of features used and mean prediction accuracy.

Predicted Outcome [Predicted Classes]	Predictive Features	Class-wise Statistics	Total # Features	Prediction Accuracy (%)
**Predicted Outcomes: Diagnoses**				

Meibomian Gland Dysfunction [No, Yes]	Visible MG: LL (#)	[15.6, 14.8]	30	74.4
	NITBUT (sec)	[13.8, 10.0]		
	Lipid Layer Thickness (nm)	[68.2, 57.8]		
	OSDI (0–100)	[13.2, 12.6]		
	CLW Hx (yrs)	[10.1, 9.9]		
Aqueous Deficiency [No, Yes]	Visible MG: LL (#)	[15.5, 14.0]	47	85.2
	Conjunctival Staining (0–3)	[1.44, 2.15]		
	Blepharitis: LL (0–3)	[0.36, 0.58]		
	VAS EOD Comfort (0–100)	[68.5, 73.25]		
	CLDEQ-8 (0–37)	[9.8, 8.0]		
Blepharitis [No, Yes]	Visible MG: UL (#)	[20.0, 17.8]	31	73.7
	Erythema: LL (0–3)	[0.18, 0.44]		
	LoM Displacement: LL (mm)	[0.72, 1.00]		
	Age (yrs)	[25.0, 30.4]		

MG = Meibomian Gland; EOD = End-of-Day; UL = Upper Lid; LL = Lower Lid; CLW = Contact Lens Wear; LoM = Line of Marx.

Fewer visible MG in the upper eyelid was also a heavily weighted predictor for a diagnosis of aqueous deficiency, along with more conjunctival staining, a higher grade of blepharitis in the lower eyelid, and interestingly a higher end-of-day comfort rating among all subjects and a lower CLDEQ-8 score among contact lens wearers (see Discussion). The prediction model for aqueous deficiency diagnosis achieved 85.2 % accuracy.

Fewer visible MG was a heavily weighted predictor for a diagnosis of blepharitis. Also heavily weighted were a higher grade of eyelid margin erythema, greater anterior displacement of the Line of Marx, and approximately 5 years greater age. The prediction model for a diagnosis of blepharitis achieved 73.7 % accuracy.

No models for predicting lagophthalmos included any MG morphological features as heavily weighted predictors.

### 3.44 predictions using only Meibomian Gland morphological features

All of the models presented above were also run using only MG morphological characteristics as predictors, that is, without any corresponding clinical signs, subjective symptoms, or subject characteristics (Table 5). In general and as expected, using gland morphology learned from meibography images as the only available features reduced prediction accuracy to a greater or lesser degree. Many clinical signs predictions that achieved high accuracies when all clinical and subjective features were available continued to be predicted with little loss of accuracy using only MG morphology. Eyelid notching was predicted with 95.4 % accuracy with a higher percentage of ghost glands as the most heavily weighted feature, compared with 95.9 % when all clinical and subjective variables were included. Similarly, erythema (97.0 % vs. 99.1 % with all features), meibum quantity (97.2 % vs. 98.0 %), corneal staining extent (90.6 % vs. 91.2 %), and Schirmer strip wetted length (91.1 % vs. 92.5 %) were all predicted by MG morphological characteristics alone with ∼2 % loss of accuracy or less. A few clinical signs were not well-predicted by gland morphology alone, including eyelid margin vascularization (62.2 % vs. 85.9 % with all features) and tear meniscus height (52.0 % vs. 72.6 % with all features).

**Table 5:** Comparison of outcome predictions with all variables available as potential predictive features vs. using MG morphological characteristics alone to make predictions. A number of clinical signs can be predicted with over 90 % accuracy using only meibography-derived MG morphological characteristics. Symptoms are generally predicted with lower accuracy than signs.

Predicted Outcome	% Accuracy - All Features	% Accuracy - MG Morphological Features Only	Most Heavily Weighted Feature
**Predicted Outcomes: Clinical Signs**			

Eyelid Notching	95.9	95.4	Higher % Ghost MG
Erythema: LL	99.1	97.0	Shorter MG Length
Eyelid Vascularization	85.9	62.2	Longer MG Length
Poor Meibum Quantity: UL, Central	98.0	97.2	Longer MG Length
Poor Meibum Quality: LL, Entire	94.0	87.4	Less Local Contrast
Greater Corneal Staining Extent	91.2	90.6	Greater % Area MG Atrophy
Lower Tear Meniscus Height	72.6	52.0	Higher % Ghost MG
Shorter FTBUT: Non-Asians	87.4	81.7	Less Local Contrast
Shorter Schirmer Strip Wetted Length	92.5	91.1	Fewer Visible Glands

**Predicted Outcomes: Subjective Symptoms**			

Higher OSDI Score	68.1	61.7	Higher % Ghost MG
Lower VAS Rating: Comfort	65.4	48.6	Higher % Ghost MG
Higher VAS Rating: Dryness	66.1	55.3	Higher % Ghost MG
DEFC Debilitating Symptoms: CL Wear	63.9	43.0	Longer MG Length
DEFC Any Symptoms: Non-CL Wear	76.8	61.9	Same MG Density
DEFC Debilitating Symptoms: Non-CL Wear	86.5	79.6	Less Tortuosity
**Predicted Outcomes: Diagnoses**			

Meibomian Gland Dysfunction	74.4	58.7	Greater % Area MG Atrophy
Aqueous Deficiency	85.2	79.5	Shorter MG Length
Blepharitis	73.7	58.2	Fewer Visible Glands

MG = Meibomian Gland; LL = Lower Lid; UL = Upper Lid; CL = Contact Lens.

Symptom predictions achieved in general lower accuracies than did clinical signs predictions, both in models with all clinical data available and in models using MG morphological characteristics alone. In addition, the discrepancy in accuracy between these two types of model is greater for symptom predictions than for signs predictions (Table 5). The highest prediction accuracies for any symptom outcomes were for presence of any DE symptoms and of debilitating symptoms using the DEFC. Prediction accuracies using MG morphological characteristics alone vs. with all clinical variables available were 61.9 % vs. 76.8 % for any DE symptoms, and 79.6 % vs. 86.5 % for debilitating DE symptoms.

Diagnoses using only MG morphological features as predictors also suffered fairly large reductions in accuracy compared with having all clinical and subjective variables available as predictors. MGD prediction accuracy was reduced from 74.4 % to 58.7 %, and relied most heavily on % area of gland atrophy. Aqueous deficiency prediction accuracy was reduced from 85.2 % to 79.5 %, with shorter gland length as the most heavily weighted feature. Blepharitis prediction accuracy was reduced from 73.7 % to 58.2 % with fewer visible glands being the most heavily weighted feature. For a diagnosis of lagophthalmos, when all clinical and subjective variables were available for prediction, the model did not rely on any MG morphological characteristic as a heavily weighted feature. When forced to predict lagophthalmos based only on gland morphology, the model achieved 68.9 % accuracy with shorter gland length as the most heavily weighted feature. However, the class-wise statistics revealed virtually no clinically recognizable differences in gland length between outcome classes (i.e., mean gland lengths for those with and without lagophthalmos were 7.28 mm and 7.29 mm, respectively).

It is important to highlight that MG morphology alone was able to predict gland function with accuracies ranging from 87 % to 99 %. However, different gland morphological characteristics emerged as the most heavily weighted predictors for meibum quality vs. quantity. For meibum quality, upper and lower eyelid scores were predicted with 94 % and 96 % accuracy, respectively. The most heavily weighted features for meibum quality were longer and wider glands with greater density, more tortuosity, fewer ghost glands, and greater contrast. For meibum quantity, upper and lower eyelid scores were predicted with 97 % and 99 % accuracy, respectively. The most heavily weighted features for meibum quantity were longer and wider glands, less area of gland atrophy, fewer ghost glands, and more visible glands.

## 4 Discussion

This work presents a novel machine learning approach to investigating the connections between MG morphology, clinical signs, subjective symptoms, and diagnoses relating to MGD and DE. A machine learning model was developed to combine meibography with an array of clinical, laboratory, and subjective symptom variables to generate predictions of MGD- and DE-related outcomes. A number of MG morphological characteristics are shown to be heavily weighted predictors of gland function, clinical signs, subjective symptoms, and clinician diagnoses. A common limitation of machine learning models for research discovery has been the difficulty in interpreting what features the model is relying on most heavily to make predictions (the “black box” problem) [31]. This algorithm design addresses the problem in that the model output includes feature weights and class-wise statistics that can allow the clinician scientist to interpret the features used by the model (with some caveats, discussed below).

The models presented here predict clinical signs with accuracies ranging from 72.6 % to 99.1 %. Prediction of subjective symptoms is more modest with accuracies ranging from 60.7 % to 86.5 %. This is presumably due to the fact that symptoms are the ultimate result of multiple factors, including loss of tear film homeostasis, hyperosmolarity, inflammation, and ultimately recruitment of ocular sensory neurons to elicit a neural signal that manifests as idiosyncratic subjective symptoms [32]. Diagnoses were predicted for MGD, aqueous deficiency, and blepharitis with 74.4 %, 85.2 %, and 73.7 % accuracies, respectively. The multifaceted nature of clinical diagnosis makes machine learning prediction more difficult than prediction of specific signs.

### 4.1 Clinical Significance of Meibomian Gland morphology

This work demonstrates the important clinical implications of MG morphology. Prior works that have attempted to study the associations of gland morphology with clinical signs and subjective symptoms have relied primarily on visual assessments of the gland atrophy area, which are limited in detail and can be susceptible to subjective judgment. This work employs a trained and validated deep learning model to quantitatively analyze detailed MG morphological characteristics on large numbers of meibography images without additional human intervention. Machine learning-derived morphological features were among the most heavily weighted predictors for conditions of the eyelids (notching, erythema, vascularization), MG function (expressate quality and quantity), tear volume and tear film stability (meniscus height, FTBUT, Schirmer strip wetted length), ocular surface health (corneal staining extent), and symptoms (OSDI, VAS comfort and dryness ratings, DEFC symptoms). The detailed morphological analysis of global and local features using this machine learning approach shows that MG morphology is indeed important to ocular surface health.

Fewer visible MG was a heavily weighted feature in predictions of MGD, aqueous deficiency and blepharitis. For MGD diagnosis, in addition to the number of visible glands, non-invasive tear breakup time and tear lipid layer thickness have clinically significant effect sizes with approximately 4 s and 10 nm differences, respectively, between MGD and non-MGD. This result supports a pathway from MG morphology to healthy gland function and an adequately thick lipid layer to inhibit tear aqueous evaporation and maintain tear film stability. It is of note that the model strongly weighted NITBUT in the prediction of MGD and did not include the more commonly used FTBUT, suggesting that FTBUT may not be as sensitive a measure of MGD-associated differences in tear film stability. This is plausible given that FTBUT is likely measuring either fluorescence quenching or reduced fluorescence intensity in the aqueous tears as the tear film thins due to evaporation and divergent flow [33] whereas NITBUT measures the optical distortion of reflected mires from the surface of the tear film [34] where any effects of an unstable or inadequate lipid layer would first be thought to manifest. That lipid layer thickness was also heavily weighted by the model in predicting MGD supports previous studies that have shown tear lipid layer thickness to impact tear film stability [[35], [36], [37], [38], [39]]. Finally, it should be noted that age was not a heavily weighted predictor of MGD. MGD has traditionally been viewed from a clinical perspective as a disease of the aging eye [40], however there is emerging evidence for significant morphological changes to the MG across age groups. For example, MGD occurs in young individuals who spend substantial time near-focused on digital devices which leads to a sub-normal blink rate and a reduction in the periodic stimulation of the glands and refreshment of the tear lipid layer [41]. This work supports the emerging consensus that MGD should not be viewed by clinicians strictly as a disease of the aging eye [42], and in cases of younger patients with DE-related symptoms and tear film instability a MG evaluation should be considered. It is important to note that despite the fairly wide age range of subjects in this study, a large majority were under the age of 35 yrs and relatively few subjects were older than 65 yrs. Further study in an older population may shed additional light on the impact of age on MGD.

A diagnosis of aqueous deficiency was predicted with 85 % accuracy by fewer visible MG and more lissamine green conjunctival staining as heavily weighted predictors. FTBUT and corneal staining assessed with fluorescein were not heavily weighted predictors of aqueous deficiency. The diagnostic value of the two dyes differs: fluorescein dye highlights corneal and conjunctival epithelial cellular disruptions while lissamine green dye highlights dead and devitalized cells. It has been shown that interferon-γ expression in the conjunctiva is upregulated in aqueous deficient DE subjects, which is correlated with increased conjunctival goblet cell loss [43]. Of interest, aqueous deficiency was predicted by a better end-of-day VAS comfort rating, and by a lower CLDEQ-8 score (less dryness) among contact lens wearers. It has been shown that many patients with aqueous deficient DE experience less severe disease symptoms due to decreased corneal nerve density and corneal desensitization induced by contact lens wear [[44], [45], [46], [47]].

Fewer visible MG was a heavily weighted predictor of a diagnosis of blepharitis, as was greater Line of Marx anterior displacement, higher grade of eyelid margin erythema, and greater age. Anterior displacement of the Line of Marx has been shown to be an indicator of the chronicity of MGD and DE [[48], [49], [50]]. Although Line of Marx displacement was not among the most heavily weighted predictors for MGD, this study provides novel evidence of this parameter being an indicator for the presence of blepharitis. That age was also a heavily weighted predictor for blepharitis raises the question of whether aging has a significant influence on the microflora of the eyelid and ocular surface, particularly at the eyelid margin. It is known that the immune and inflammatory responses to alterations of the body's microflora change with age [51].

This study shows that machine learning-derived MG morphological characteristics alone can predict several individual clinical signs but cannot predict diagnoses of MGD and blepharitis without input from clinical assessments. This is in line with results in the literature that are equivocal with respect to gland atrophy measured by meiboscore being a risk factor for MGD. It is also of interest that the prediction model did not utilize more years of contact lens wear as a heavily weighted predictor for MGD. Evidence for the role of contact lens wear in MGD in the literature has also been equivocal [52].

### 4.2 Limitations and interpretation in the clinical setting

One of the goals of this investigation was to be able to make scientific and clinical interpretations of the most heavily weighted predictive features for a given outcome. It must be remembered in interpreting the output that these models have no way of establishing a direction of causality. That can only be established through prospective, longitudinal investigations and scientific consensus. It must also be remembered that employing machine learning in this setting does not completely mitigate human bias. The algorithm solves a classification problem in which the predicted classes are human-defined based on published thresholds and clinical experience. Furthermore, human judgment is the source of all clinical assessment grades upon which the algorithm operates. Finally, as with any human research investigation, the results depend upon the characteristics of the study population from which subjects were sampled. The models in this study were trained on data from a single site, located in the ethnically diverse San Francisco Bay Area, and centered around the University of California Berkeley campus with its attendant younger demographics. Given the above limitations, we believe it to be imperative with the rapidly emerging role of artificial intelligence in health care and biomedical research that these powerful machine learning approaches be used as complementary tools to expert clinical judgement, and not overly relied upon as “black-box” definitive diagnostics.

With respect to interpretation of these results in the clinical care setting, the number of visible glands was a heavily weighted predictive feature for 5 different clinical signs, 3 symptom assessments, and all 3 diagnoses. MG width was predictive of meibum quantity and gland local contrast was predictive of meibum quality. Together these results suggest that having as many glands as possible, and having plump (greater gland width) and bright (high local contrast) glands is optimal for ocular surface health. In agreement with some previous work [53,54], the results presented here suggest that more MG tortuosity might not be pathological, and in fact was associated with a lower grade of eyelid margin erythema and with having a DEFC classification of asymptomatic for DE. While it appears abnormal in the meibography image, in fact a tortuous gland is longer and presumably produces more meibum than a straight gland. Provided the glands are plump and bright in the image, an observation of significant tortuosity may not indicate pathology.

## 5 Conclusions

In this study a novel machine learning-based method that permits clinical interpretation of the relationships between MG morphology and clinical outcomes was developed. Analyzing the relationships of signs, symptoms and diagnoses to MG morphology has contributed novel observations relating to MGD and DE. In addition, the associations of MG morphology with signs, symptoms, and diagnoses provides clear evidence that gland morphology encodes much more information on the health of the anterior eye than previously thought.

## Grant/financial support

UCB-CRC Unrestricted Fund (MCL), Roberta J. Smith Research Fund (MCL), R21EY033881 (MCL & SXY), T32EY007043 (VT). The funding organizations had no role in the design or conduct of this research.

## CRediT authorship contribution statement

**Andrew D. Graham:** Writing – review & editing, Writing – original draft, Visualization, Validation, Supervision, Methodology, Investigation, Data curation, Conceptualization. **Tejasvi Kothapalli:** Validation, Software, Methodology, Investigation, Formal analysis. **Jiayun Wang:** Validation, Software, Methodology, Investigation, Formal analysis. **Jennifer Ding:** Visualization, Investigation, Data curation. **Vivien Tse:** Visualization, Methodology, Investigation, Data curation. **Penny A. Asbell:** Writing – review & editing, Resources, Investigation. **Stella X. Yu:** Supervision, Methodology, Investigation, Funding acquisition, Formal analysis, Conceptualization. **Meng C. Lin:** Writing – review & editing, Writing – original draft, Visualization, Supervision, Resources, Project administration, Methodology, Investigation, Funding acquisition, Conceptualization.

## Declaration of competing interest

There are no conflicts of interest for any author. All photographic images were taken by the authors.
