# Supplementary material for: A machine learning approach to predicting dry eye-related signs, symptoms and diagnoses from meibography images
Source: Heliyon. 2024 Aug 13;10(17):e36021. doi: 10.1016/j.heliyon.2024.e36021 (PMC11403426; doi:10.1016/j.heliyon.2024.e36021)
Supplement: Multimedia component 1 [file mmc1.pdf]

UCB_CRC_OS4 Study: OCULAR SURFACE DISEASE INDEX

Study Code	Investigator	Exam Date (mm/dd/yy)	Visit #	Subject ID	Unique ID
OS4		/ /			

For office use only: 1

Have you experienced any of the following during the last week?

	All of the time	Most of the time	Half of the time	Some of the time	None of the time
Eyes that are sensitive to light?	4	3	2	1	0
Eyes that feel gritty?	4	3	2	1	0
Painful or sore eyes?	4	3	2	1	0
Blurred vision?	4	3	2	1	0
Poor vision?	4	3	2	1	0

For office use only: Subtotal:

Have problems with your eyes limited you in performing any of the following during the last week?

	All of the time	Most of the time	Half of the time	Some of the time	None of the time	
Reading?	4	3	2	1	0	N/A
Driving at night?	4	3	2	1	0	N/A
Working with a computer or bank machine (ATM)?	4	3	2	1	0	N/A
Watching TV?	4	3	2	1	0	N/A

For office use only: Subtotal:

Have your eyes felt uncomfortable in any of the following situations during the last week?

	All of the time	Most of the time	Half of the time	Some of the time	None of the time	
Windy conditions?	4	3	2	1	0	N/A
Places or areas with low humidity (very dry)?	4	3	2	1	0	N/A
Areas that are air conditioned?	4	3	2	1	0	N/A

For office use only: Subtotal:

For office use only: Calculated OSDI Score: .

(Total Sum x 25 / # of questions answered, not including N/A)

61050

UCB_CRC_OS4 STUDY: DISCOMFORT (VAS) QUESTIONNAIRE - AVERAGE

Study Code	Investigator	Exam Date (mm/dd/yy)	Visit #	Subject ID	Unique ID	For office use only: 3a
O S 4		/ /				

Please place a vertical line (|) through each scale below to indicate the **AVERAGE DAILY DISCOMFORT** that you experience in your eyes.

1) How would you rate the **average daily discomfort** of each eye?

RIGHT EYE:

No discomfort whatsoever — Extreme discomfort, intolerable

LEFT EYE:

No discomfort whatsoever — Extreme discomfort, intolerable

2) **On average** during the day, how **often** do you experience **discomfort** in each eye?

RIGHT EYE:

Never — All the time

LEFT EYE:

Never — All the time

UCB_CRC_OS4 STUDY: DRYNESS (VAS) QUESTIONNAIRE - AVERAGE

Study Code	Investigator	Exam Date (mm/dd/yy)	Visit #	Subject ID	Unique ID
OS4		/ /			

For office use only: 3b

Please place a vertical line (|) through each scale below to indicate the **AVERAGE DAILY DRYNESS** that you experience in your eyes.

1) How would you rate the **average daily dryness** of each eye?

RIGHT EYE:

No sensation of dryness whatsoever — Extremely dry, intolerable

LEFT EYE:

No sensation of dryness whatsoever — Extremely dry, intolerable

2) **On average** during the day, how **often** do you experience **dryness** in each eye?

RIGHT EYE:

Never — All the time

LEFT EYE:

Never — All the time

UCB_CRC_OS4 STUDY: DISCOMFORT (VAS) QUESTIONNAIRE - EOD

Study Code	Investigator	Exam Date (mm/dd/yy)	Visit #	Subject ID	Unique ID
O S 4		/ /			

For office use only:
3c

Please place a vertical line (|) through each scale below to indicate the **DISCOMFORT** that you experience in your eyes **AT THE END OF THE DAY**.

1) How would you rate the **discomfort** of each eye **at the end of the day**?

RIGHT EYE:

No discomfort whatsoever ———————————— Extreme discomfort, intolerable

LEFT EYE:

No discomfort whatsoever ———————————— Extreme discomfort, intolerable

2) How **often** do you experience **discomfort** in each eye **at the end of the day**?

RIGHT EYE:

Never ———————————— All the time

LEFT EYE:

Never ———————————— All the time

UCB_CRC_OS4 STUDY: DRYNESS (VAS) QUESTIONNAIRE - EOD

Study Code	Investigator	Exam Date (mm/dd/yy)	Visit #	Subject ID	Unique ID
O S 4		/ /			

For office use only:
3d

Please place a vertical line (|) through each scale below to indicate the **DRYNESS** that you experience in your eyes **AT THE END OF THE DAY**.

1) How would you rate the **dryness** of each eye **at the end of the day**?

RIGHT EYE:

No sensation of dryness whatsoever — Extremely dry, intolerable

LEFT EYE:

No sensation of dryness whatsoever — Extremely dry, intolerable

2) How **often** do you experience **dryness** in each eye **at the end of the day**?

RIGHT EYE:

Never — All the time

LEFT EYE:

Never — All the time

55334

UCB_CRC_OS4 STUDY: CLW DRY EYE FLOW CHART

Study Code	Investigator	Exam Date (mm/dd/yy)	Visit #	Subject ID	Unique ID
O S 4		/ /			

For office use only: 5b

Do you experience dryness in your eyes while wearing contact lenses? ***Check one box only.***

☐ No

☐ Yes

Does the dryness you experience make you uncomfortable? ***Check one box only.***

☐ No

☐ Yes

How often would you say the dryness you experience interferes with your activities (reading, using computer, wearing contact lenses)? ***Check one box only.***

☐ Never/Rarely

☐ Sometimes

☐ Usually/Always

22119

UCB_CRC_TEAR4 STUDY: NON-CLW DRY EYE FLOW CHART

Study Code	Investigator	Exam Date (mm/dd/yy)	Visit #	Subject ID	Unique ID	For office use only:
TEAR4		/ /				5

Do you experience dryness in your eyes? ***Check one box only.***

→ ☐ No

☐ Yes ↓

Does the dryness you experience make you uncomfortable? ***Check one box only.***

→ ☐ No

☐ Yes ↓

How often would you say the dryness you experience interferes with your activities (reading or using the computer)? ***Check one box only.***

→ ☐ Never/Rarely

→ ☐ Sometimes

→ ☐ Usually/Always

UCB_CRC_OS4 STUDY: SPEED II Questionnaire

Study Code	Investigator	Exam Date (mm/dd/yy)	Visit #	Subject ID	Unique ID
OS4		/ /			

For office use only:
6

1A. Report the **FREQUENCY** of dry eye symptoms you are experiencing by checking Never, Sometimes, Often, or Constant using the numbering system below:

	0	1	2	3
**Dryness, Grittiness, or Scratchiness**				
**Soreness or Irritation**				
**Burning or Watering**				
**Eye Fatigue**				

0 = Never
1 = Sometimes
2 = Often
3 = Constant

1B. Are your symptoms worse in one eye? ☐ **No** ☐ **Yes** If **YES,** right or left? ☐ **Right** ☐ **Left**

2A. Report the **SEVERITY** of dry eye symptoms you are experiencing by checking Never, Sometimes, Often, or Constant using the numbering system below:

	0	1	2	3	4
**Dryness, Grittiness, or Scratchiness**					
**Soreness or Irritation**					
**Burning or Watering**					
**Eye Fatigue**					

0 = No problems
1 = Tolerable- not perfect but not comfortable
2 = Uncomfortable- irritating but does not interfere with my day
3 = Bothersome- irritating and interferes with my day
4 = Intolerable- unable to perform my daily tasks

2B. Are your symptoms worse in one eye? ☐ **No** ☐ **Yes** If **YES,** right or left? ☐ **Right** ☐ **Left**

3. Please mark with an X if you have experienced symptoms:
☐ **Today** ☐ **Within the past 72 hours** ☐ **Within the past 3 months**

4. Do you have fluctuating vision problems? (That can be corrected with blinking)
☐ **Never** ☐ **Sometimes** ☐ **Frequently** ☐ **A Lot/Always**

5. Have you been told that you have blepharitis or have been treated for a stye?

Blepharitis ☐ **Yes** ☐ **No**
Stye ☐ **Yes** ☐ **No**

6. NOTE: Please add any additional questions, comments, and/or concerns you may wish to share.

For office use only:
Total Speed Score (Frequency + Severity) =

21678

Study Code	Investigator	Exam Date (mm/dd/yy)	Visit #	Subject ID	Unique ID	For office use only: 8a
O S 4		/ /				

CONTACT LENS QUESTIONNAIRE-8 (CLDEQ-8)

Please circle the number of your response to each question:

1. Questions about **EYE DISCOMFORT**:

   a. During a typical day in the past 2 weeks, **how often** did your eyes feel discomfort while wearing your contact lenses?

   0 Never
   1 Rarely
   2 Sometimes
   3 Frequently
   4 Constantly

   When your eyes felt discomfort with your contact lenses, **how intense was this feeling of discomfort ...**

   b. At the end of your wearing time?

Never have it	Not at All Intense				Very Intense
0	1	2	3	4	5

2. Questions about **EYE DRYNESS:**

   a. During a typical day in the past 2 weeks, **how often** did your eyes feel dry?

   0 Never
   1 Rarely
   2 Sometimes
   3 Frequently
   4 Constantly

   When your eyes felt dry, **how intense was this feeling of dryness...**

   b. At the end of your wearing time?

Never have it	Not at All Intense				Very Intense
0	1	2	3	4	5

For office use only:
Sum of scores: [ ][ ]

3. Questions about **CHANGEABLE, BLURRY VISION:**

   a. During a typical day in the past 2 weeks, **how often** did your vision change between clear and blurry or foggy while wearing your contact lenses?

   0 Never
   1 Rarely
   2 Sometimes
   3 Frequently
   4 Constantly

   When your vision was blurry, **how noticeable was the changeable, blurry, or foggy vision ...**

   b. At the end of your wearing time?

Never have it	Not at All Intense				Very Intense
0	1	2	3	4	5

4. Questions about **CLOSING YOUR EYES:**
   During a typical day in the past 2 weeks, how often did your **eyes bother you so much that you wanted to close them**?

   0 Never
   1 Rarely
   2 Sometimes
   3 Frequently
   4 Constantly

5. Questions about **REMOVING YOUR LENSES:**
   How often during the past 2 weeks, did your eyes *bother you so much* while wearing your contact lenses that you felt as if you needed to stop whatever you were doing and **take out your contact lenses**?

   1 Never
   2 Less than once a week
   3 Weekly
   4 Several times a week
   5 Daily
   6 Several times a day

UCB_CRC_TEAR4 STUDY: DEQ-5

Study Code	Investigator	Exam Date (mm/dd/yy)	Visit #	Subject ID	Unique ID	For office use only:
TEAR4		/ /				7

DEQ-5

Please circle the number of your response to each question:

1. Questions about **EYE DISCOMFORT**:
   a. During a typical day in the past month, **how often** did your eyes feel discomfort?
      0 Never
      1 Rarely
      2 Sometimes
      3 Frequently
      4 Constantly

   b. When your eyes felt discomfort, **how intense was this feeling of discomfort** at the end of the day, within two hours of going to bed?

Never have it	Not at All Intense				Very Intense
0	1	2	3	4	5

2. Questions about **EYE DRYNESS:**
   a. During a typical day in the past month, **how often** did your eyes feel dry?
      0 Never
      1 Rarely
      2 Sometimes
      3 Frequently
      4 Constantly

   b. When your eyes felt dry, **how intense was this feeling of dryness** at the end of the day, within two hours of going to bed?

Never have it	Not at All Intense				Very Intense
0	1	2	3	4	5

3. Questions about **WATERY EYES:**

   During a typical day in the past month, **how often** did your eyes look or feel excessively watery?
      0 Never
      1 Rarely
      2 Sometimes
      3 Frequently
      4 Constantly

For office use only:
Sum of scores: ☐☐

29268
